# Exenatide Is an Effective Antihyperglycaemic Agent in a Mouse Model of Wolfram Syndrome 1

**DOI:** 10.1155/2016/9239530

**Published:** 2016-03-16

**Authors:** Tuuli Sedman, Kertu Rünkorg, Maarja Krass, Hendrik Luuk, Mario Plaas, Eero Vasar, Vallo Volke

**Affiliations:** ^1^Department of Physiology, Institute of Biomedicine and Translational Medicine, University of Tartu, 19 Ravila Street, 50411 Tartu, Estonia; ^2^Centre for Translational Medicine, Institute of Biomedicine and Translational Medicine, University of Tartu, 19 Ravila Street, 50411 Tartu, Estonia; ^3^Laboratory Animal Centre, Institute of Biomedicine and Translational Medicine, University of Tartu, 19 Ravila Street, 50411 Tartu, Estonia; ^4^Tartu University Hospital, 8 L. Puusepa Street, 51014 Tartu, Estonia

## Abstract

Wolfram syndrome 1 is a very rare monogenic disease resulting in a complex of disorders including diabetes mellitus. Up to now, insulin has been used to treat these patients. Some of the monogenic forms of diabetes respond preferentially to sulphonylurea preparations. The aim of the current study was to elucidate whether exenatide, a GLP-1 receptor agonist, and glipizide, a sulphonylurea, are effective in a mouse model of Wolfram syndrome 1. Wolframin-deficient mice were used to test the effect of insulin secretagogues. Wolframin-deficient mice had nearly normal fasting glucose levels but developed hyperglycaemia after glucose challenge. Exenatide in a dose of 10 *μ*g/kg lowered the blood glucose level in both wild-type and wolframin-deficient mice when administered during a nonfasted state and during the intraperitoneal glucose tolerance test. Glipizide (0.6 or 2 mg/kg) was not able to reduce the glucose level in wolframin-deficient animals. In contrast to other groups, wolframin-deficient mice had a lower insulin-to-glucose ratio during the intraperitoneal glucose tolerance test, indicating impaired insulin secretion. Exenatide increased the insulin-to-glucose ratio irrespective of genotype, demonstrating the ability to correct the impaired insulin secretion caused by wolframin deficiency. We conclude that GLP-1 agonists may have potential in the treatment of Wolfram syndrome-related diabetes.

## 1. Introduction

Wolfram syndrome 1 is a very rare monogenic disease resulting in a complex of disorders including diabetes mellitus, diabetes insipidus, optic atrophy, and hearing loss [1]. The affected gene (*Wfs1*) encodes wolframin protein [2], which is involved in the machinery of the endoplasmatic reticulum but its definite function remains obscure.

Diabetes is one of the hallmarks of Wolfram syndrome. Diabetes is present in almost all affected individuals and is usually one of the earliest abnormalities diagnosed [3]. In recent patient series, the median onset of diabetes has been between 6.3 and 8.1 years [3, 4]. Wolfram syndrome-related diabetes (WSD) is insulin-dependent, but with an earlier presentation and slower progression than classical type 1 diabetes [5]. Moreover, variants of the wolframin gene may increase the risk of type 2 diabetes [6, 7].

WSD is currently classified as one of the monogenic forms of diabetes [8]. Monogenic forms of diabetes are a heterogeneous group, but some of them respond preferentially to therapy with sulphonylurea preparations and are among the few examples where truly personalized therapy is possible in diabetes patients. Monogenic diabetes is an interesting model to study the mechanisms of glucose metabolism, and therefore animal models resembling the disorders have been developed including glucokinase-deficient mice [9]. Several groups have developed and characterized mouse models of Wolfram syndrome 1 [10–12].

Wolframin-deficient mice develop diabetes; however, the phenotype has been slightly different between various knockout lines [10–12].

Up to now, there have been no specific treatment recommendations, and, thus, insulin has been used to treat WSD in patients. Bearing in mind that insulin loss is gradual in the case of WSD and that some monogenic forms of diabetes are sensitive towards insulin secretagogues, the aim of the current study was to elucidate whether exenatide, a GLP-1 receptor agonist, and glipizide, a sulphonylurea, are effective glucose-lowering agents in a mouse model of Wolfram syndrome 1.

## 2. Materials and Methods

### 2.1. Drugs and Chemicals

Commercially available exenatide solution (Byetta, Eli Lilly, Houten, Netherlands) was used. Exenatide was diluted in saline (154 mmol/L NaCl) and injected subcutaneously in a dose of 10 *μ*g/kg. The dose of exenatide was selected according to our previous studies [13, 14]. Glipizide (Sigma-Aldrich, Missouri, USA) was dissolved in a few drops of DMSO and then diluted to the final concentration using saline. Glipizide was injected intraperitoneally in a dose of 0.6 or 2 mg/kg. In the intraperitoneal glucose tolerance test, a 20% glucose solution was used (Braun, Melsbungen, Germany) in a dose of 2 g/kg. All injections were carried out in a volume of 10 mL/kg.

### 2.2. Experimental Animals

Wfs1-deficient mice (*Wfs1* ^*− / −*^), their wild-type (*Wfs1* ^*+/+*^), and heterozygous (*Wfs1* ^*+/ −*^) littermates were used. Wfs1-deficient mice were F2 hybrids ([129S6/SvEvTac × C57BL/6] × [129S6/SvEvTac × C57BL/6]) [15] and breeding and genotyping were performed in the Department of Physiology, Institute of Biomedicine and Translational Medicine, University of Tartu. Male mice aged 6-7 months weighing 17–26 g were used. The mice were kept, 8 per cage, in an animal house under standard conditions (temperature 20–22°C, 12/12 h light-dark cycle) with free access to food and water (unless otherwise stated). All the animal procedures described in this study were conducted according to the* Guide for The Care and Use of Laboratory Animals*, eighth edition (2011), and had the permission of the Estonian National Committee for Ethics in Animal Experimentation (number 13, June 16, 2009).

### 2.3. Animal Procedures

#### 2.3.1. Experiment I: Acute Effects of Exenatide and Glipizide on Nonfasting Glucose Level

Exenatide was injected s.c. 90 minutes before the glucose measurement, and glipizide was injected i.p. 60 minutes before the measurement. All groups consisted of 8 animals.

#### 2.3.2. Experiment II: Acute Effect of Exenatide on Glucagon Secretion

Glucose was measured and blood samples for insulin were taken before and 60 minutes after exenatide injection; after that, animals were killed and the blood samples for glucagon were collected. All groups consisted of 8 animals.

#### 2.3.3. Intraperitoneal Glucose Tolerance Test (IPGTT)

The mice were fasted overnight for 12 h. A glucose solution (2 g/kg) was administered by intraperitoneal injection (at time point 0 min). Exenatide (10 *μ*g/kg, s.c.), glipizide (0.6 mg/kg or 2 mg/kg, i.p.), or saline was injected 30 minutes before the glucose administration (time point −30). Blood glucose was monitored at time points −30, 0, 15, 30, 60, and 120 minutes. Blood for insulin measurements was collected at time points −30 and 60 minutes by tail bleed. All groups consisted of 8 animals.

### 2.4. Biochemistry

Plasma insulin levels were determined using a mouse insulin ELISA kit (Crystal Chem, Illinois, US). Plasma glucagon levels were determined using a mouse glucagon ELISA kit (Kamiya Biomedical Company, Washington, USA). The samples were assayed in duplicate. All manufacturer guidelines were observed. Glucose concentration in blood was determined using an Abbott Optium Xceed glucometer (Abbott Diabetes Care, Alameda, CA).

### 2.5. Statistical Methods

Two-way ANOVA was conducted to test for effects of genotype and treatment. To compare multiple time points, ANOVA with time as repeated measure was used. The Duncan post hoc test was used afterwards to test for differences between specific groups and conditions. Data are presented as mean ± SEM. A *p* value of < 0.05 was considered statistically significant. For the statistical analysis, STATISTICA 7 (StatSoft, Bedford, UK) was used. Area under the curve was calculated using the trapezoidal method using GraphPad Prism 6 (GraphPad Software Inc., California, US).

## 3. Results

### 3.1. Acute Stress Induces Hyperglycaemia in Wfs1-Deficient Mice

**Figure 1:**
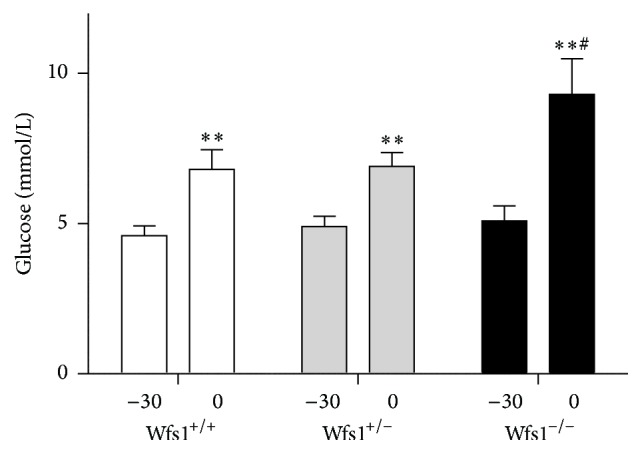
Stress-induced hyperglycaemia before IPGTT. Wfs1^+/+^, white bars; Wfs1^+/−^, grey bars; Wfs1^−/−^, black bars. Repeated measures ANOVA, followed by the Duncan post hoc test, where ^*∗∗*^ *p* < 0.01 versus time point −30; ^#^ *p* < 0.05 versus other genotypes at time point 0. Data of saline treated animals were pooled from two independent experiments. *n* = 16 in all groups.

During the intraperitoneal glucose tolerance test, all genotypes irrespective of treatment group experienced a certain hyperglycaemia during the first 30 minutes as a response to an acute stress (handling, blood sample collection, and injection). ANOVA with time as repeated measure indicated a significant effect of time (*F* = 48; *p* < 0.001) and close to a significant interaction of genotype × time (*F* = 2.9; *p* = 0.068). The post hoc Duncan test revealed that glucose levels were significantly higher at the time point of 30 min in all groups, and Wfs1-deficient mice had augmented hyperglycaemia compared to heterozygotes (*p* < 0.05) or their wild-type littermates (*p* < 0.05) (Figure 1).

### 3.2. Exenatide Lowers Blood Glucose Level in Wfs1-Deficient Mice and Glipizide Has No Significant Effect

**Figure 2:**
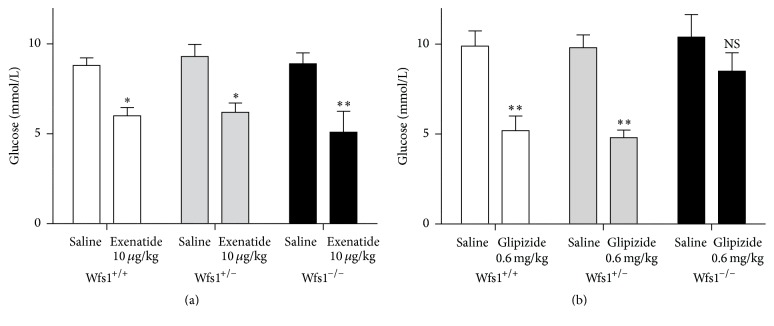
Effect of treatment with exenatide (a) and glipizide (b) on blood glucose level. Wfs1^+/+^, white bars; Wfs1^+/−^, grey bars; Wfs1^−/−^, black bars. Two-way ANOVA was used, followed by Duncan post hoc test, where ^*∗*^ *p* < 0.01 versus saline; ^*∗∗*^ *p* < 0.001 versus saline. *n* = 8 in all groups.

Two-way ANOVA revealed a significant effect of treatment with exenatide on the blood level of glucose (*F* = 33.6, *p* < 0.001). An experiment with glipizide revealed significant effects of genotype (*F* = 3.5, *p* < 0.05) and treatment (*F* = 29.1, *p* < 0.001). The post hoc Duncan test revealed that exenatide lowered glucose levels compared to saline in every genotype (*p* < 0.01 in wild-type and heterozygotes, *p* < 0.001 in Wfs1-deficient mice) (Figure 2(a)). Glipizide lowered the glucose level in wild-type mice (*p* < 0.001) and heterozygotes (*p* < 0.001) but had no significant effect in Wfs1-deficient mice (*p* = 0.174) (Figure 2(b)).

### 3.3. Exenatide Has a Robust Glucose-Lowering Effect during IPGTT in Wfs1-Deficient Mice and Glipizide Has No Significant Effect

**Figure 3:**
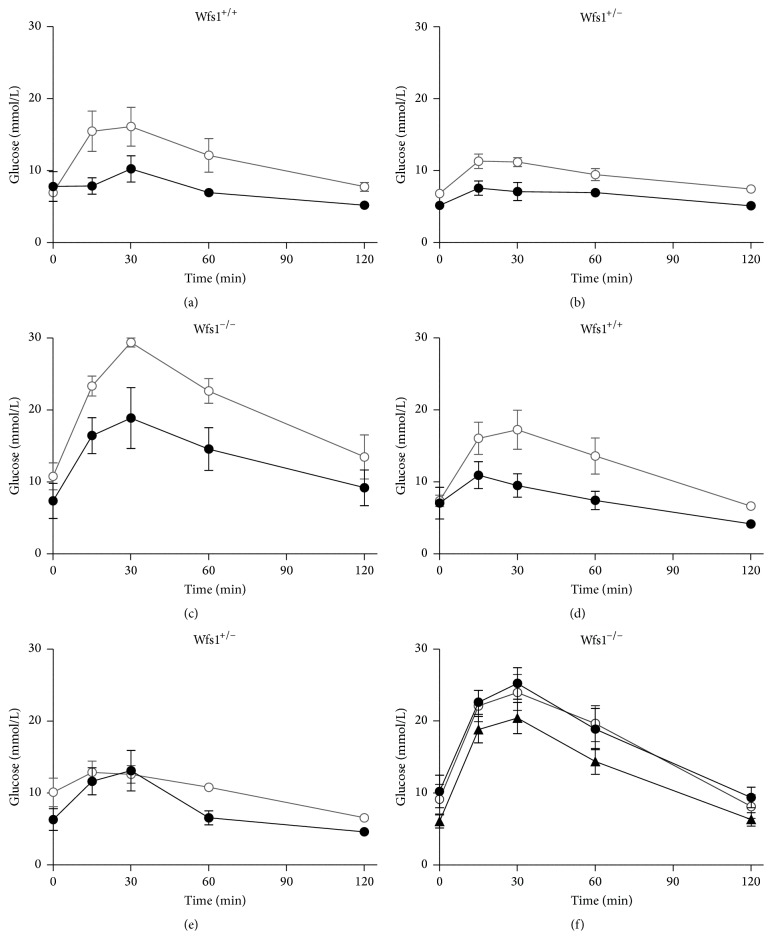
Glucose levels during IPGTT with exenatide in Wfs1^+/+^ (a), Wfs1^+/−^ (b), and Wfs1^−/−^ mice (c); saline (white dots), exenatide 10 *μ*g/kg (black dots), and glipizide in Wfs1^+/+^ (d), Wfs1^+/−^ (e), and Wfs1^−/−^ mice (f); saline (white dots), glipizide 0.6 mg/kg (black dots), and glipizide 2 mg/kg (black triangles). *n* = 8 in all groups.

Administration of glucose (2 g/kg i.p.) induced a rise in blood glucose level in all genotypes in spite of the treatment. The maximum blood level peak was traceable 30 minutes after glucose administration (Figure 3).

**Figure 4:**
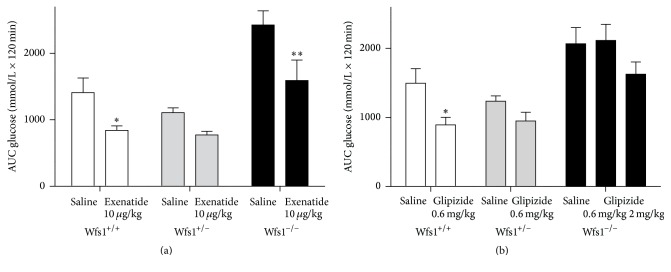
Effect of treatment with exenatide (a) and glipizide (b) on glucose AUC during IPGTT. Wfs1^+/+^, white bars; Wfs1^+/−^, grey bars; Wfs1^−/−^, black bars. Two-way ANOVA was used, followed by Duncan post hoc test, where ^*∗*^ *p* < 0.05 versus saline; ^*∗∗*^ *p* < 0.01 versus saline. *n* = 8 in all groups.

Two-way ANOVA revealed that both exenatide and glipizide had a significant genotype effect on glucose AUC during IPGTT (exenatide* F* = 19.3, *p* < 0.001; glipizide* F* = 18.9, *p* < 0.001). Treatment had a significant effect in the exenatide experiment (*F* = 14.9, *p* < 0.001) and close to a significant effect in the glipizide group (*F* = 3.7, *p* = 0.063). Treatment with exenatide lowered AUC significantly in wild-type mice (*p* < 0.05) and in Wfs1-deficient animals (*p* < 0.01), but in heterozygotes the decrease was not statistically significant (*p* = 0.233); the biggest decrease was in the Wfs1-deficient group (Figure 4(a)). Treatment with glipizide decreased AUC significantly in wild-type mice (*p* < 0.05), but in heterozygotes and the Wfs1-deficient group the effect was not significant (heterozygotes *p* = 0.268 Wfs1-deficient group glipizide 0.6 mg/kg *p* = 0.855 and glipizide 2 mg/kg *p* = 0.09) (Figure 4(b)). The higher dose of glipizide was used only in Wfs1-deficient animals.

### 3.4. Exenatide Increases Insulin-to-Glucose Ratio Irrespective of Genotype

**Figure 5:**
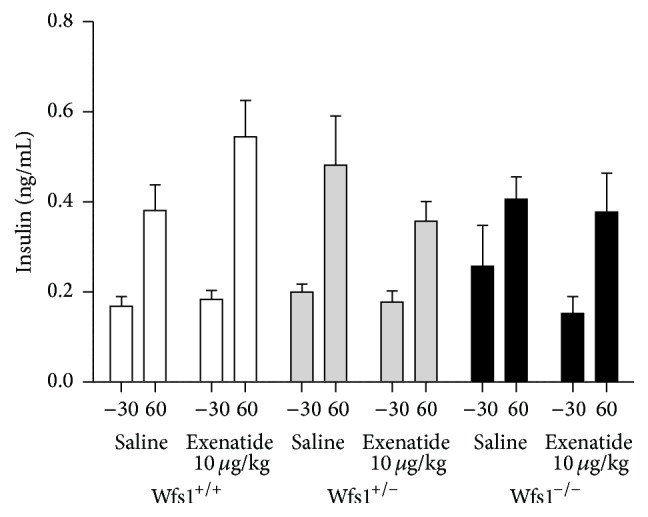
Effect of treatment with exenatide during IPGGT on insulin levels. Wfs1^+/+^, white bars; Wfs1^+/−^, grey bars; Wfs1^−/−^ black bars. To compare the insulin levels between baseline (−30 min) and the 60-minute time point, repeated measures ANOVA was used. *n* = 8 in all groups.

**Figure 6:**
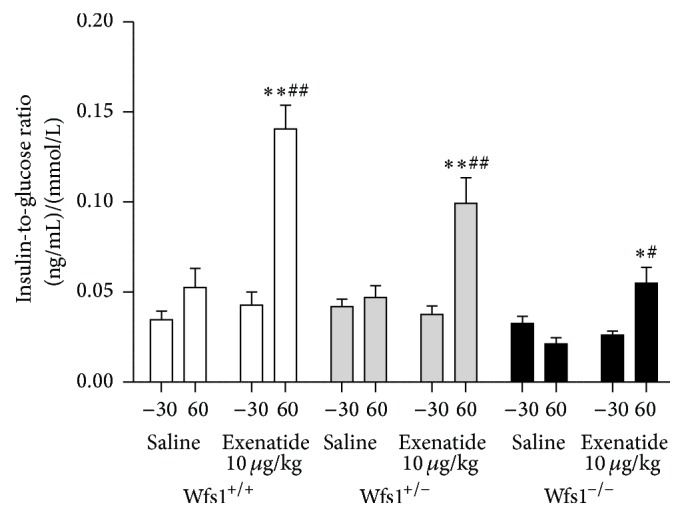
Effect of treatment with exenatide during IPGGT on insulin-to-glucose ratio. Wfs1^+/+^, white bars; Wfs1^+/−^, grey bars; Wfs1^−/−^, black bars. To compare the glucose-insulin ratio between baseline (−30 min) and the 60-minute time point, repeated measures ANOVA was used, followed by the Duncan post hoc test, where ^*∗*^ *p* < 0.05 versus baseline; ^*∗∗*^ *p* < 0.001 versus baseline. To compare the treatment effect at the 60-minute time point, two-way ANOVA was used, followed by the Duncan post hoc test, where ^#^ *p* < 0.05 versus saline at the same time point; ^##^ *p* < 0.001 versus saline at the same time point. *n* = 8 in all groups.

During the IPGTT with exenatide treatment there were no differences in basal levels of insulin. Exenatide tended to augment the rise in insulin levels but this change was not statistically significant (Figure 5). Since the glucose levels at the 60 min time point were markedly different between the groups, we calculated the insulin-to-glucose ratio. ANOVA with time as repeated measure indicated a significant effect of time × genotype × treatment with exenatide on the insulin-to-glucose ratio (*F* = 14.6, *p* < 0.001). In contrast to other groups, wolframin-deficient mice had a lower insulin-to-glucose ratio during the IPGTT, indicating impaired insulin secretion. Exenatide increased the insulin-to-glucose ratio in all genotypes (*p* < 0.05 in every group; Figure 6).

### 3.5. Exenatide Had No Significant Effect on Glucagon Level

ANOVA indicated no effect of treatment or genotype on glucagon level. Sixty minutes after the treatment, the glucagon level was comparable in all groups irrespective of treatment or genotype (Table 1).

## 4. Discussion

We have further described the diabetic phenotype of mice lacking a functional wolframin gene.

The perturbation of glucose metabolism was apparent only in homozygous mice. Mice with one copy of the functional wolframin gene had quite similar characteristics to control mice in all experiments.

Similarly to a previous report, wolframin-deficient mice had nearly normal fasting glucose levels but developed hyperglycaemia after glucose challenge [10].

Interestingly, fasted wolframin-deficient mice displayed a clearly augmented hyperglycaemic response 30 minutes after relatively mild stress, blood sampling by tail bleed and subcutaneous injection of saline. This kind of stress-induced hyperglycaemia has been previously demonstrated in another diabetes model, ob/ob mice [16]. However, in the previous report a much more stressful protocol (30 min of immobilization followed by 5 min of shaking) was used. It has been previously shown that wolframin-deficient mice display an exaggerated corticosterone response [17]. Thus, we speculate that the stress-induced hyperglycaemia results from a higher corticosterone response to stress in combination with limited availability of insulin.

We next characterized the effects of GLP-1 receptor agonist and sulphonylurea on glucose regulation. As expected, both exenatide (10 *μ*g/kg s.c.) and glipizide (0.6 mg/kg i.p.) significantly decreased glucose levels in control mice and in heterozygotes when injected in a nonfasted state. Interestingly, in wolframin-deficient mice, sulphonylurea did not change glucose levels after acute administration. In contrast, exenatide in a dose of 10 *μ*g/kg decreased glucose levels as effectively as in the case of control mice. As the first experiment was performed with food available* ad libitum*, one can argue that the effect of GLP-1 receptor agonist may, at least partly, be explained by the anorexigenic effect of the drug. Nevertheless, in the next set of experiments, the effects of drugs were studied in the intraperitoneal glucose tolerance test with fasting animals and no availability of food. The results in the IPGTT were very similar to the previous experiment: exenatide was clearly effective in wolframin-deficient mice while the glipizide effect was diminished.

Thus, one can conclude that wolframin-deficient mice display contrasting sensitivity towards different insulin secretagogues, and GLP-1 agonists may have potential in the treatment of Wolfram syndrome diabetes. Previously, pioglitazone treatment has been shown to protect wolframin-deficient mice against diabetes [18]. These data are, however, not directly comparable to ours, as the model combined wolframin deletion with the introduction of an agouti lethal yellow mutation to promote insulin resistance.

We also studied the possible mechanisms beyond the effect of GLP-1 agonist.

There was no difference in glucagon levels after exenatide administration in any group of mice.

The insulin levels in the glucose tolerance test tended to be higher after exenatide administration but the difference was not statistically significant. However, one must take into account the fact that in wolframin-deficient animals glucose levels were much higher after glucose challenge. Thus, insulin-to-glucose ratios were also calculated. In contrast to other groups, wolframin-deficient mice had a lower insulin-to-glucose ratio during the IPGTT, indicating impaired insulin secretion. Exenatide increased the insulin-to-glucose ratio during the glucose tolerance test irrespective of genotype, demonstrating the ability to correct the impaired insulin secretion caused by wolframin deficiency.

Recent evidence from other groups supports the potential effect of GLP-1 agonists in the case of Wolfram syndrome-related diabetes. One of the final effectors in the pathway after GLP-1 receptor activation is synaptotagmin-7, a molecule involved in calcium-sensitive transmitter (including insulin) release [19]. In line with that, a recent report using Wfs1-deficient beta cells as a model linked altered calcium homeostasis with cell death. Several molecules were able to protect beta cells in the aforementioned model and GLP-1 was one of them [20]. Thus, we speculate that in case of WSD there is a defect in a calcium-dependent release of insulin, which may explain why GLP-1 receptor agonist, but not sulfonylurea, was effective in the model.

Thus, we propose that GLP-1 agonists could be tried in the treatment of (early) WSD patients. GLP-1 receptor agonists have a solid background in terms of efficacy and safety in clinical studies. Exenatide has been successfully tried in an HNF-1alpha type of MODY patient [21]. The key benefits of GLP-1 receptor agonist therapy would be the lower risk of hypoglycaemia and the lower number of injections needed.

As a limitation, one has to realize that islet structure, function, and beta cell regulation is different between rodents and humans. For example, chronic treatment of mice with liraglutide led to a decrease of beta cell mass [22]. Thus, our encouraging findings in mice do not automatically guarantee clinical success in patients.

## 5. Conclusions

We conclude that wolframin-deficient mice display contrasting sensitivity towards different insulin secretagogues, and GLP-1 agonists may have potential in the treatment of Wolfram syndrome-related diabetes.

## Figures and Tables

**Table 1:** Effect of treatment with exenatide on glucagon level.

Genotype	Treatment	Glucagon (ng/mL)
Wild-type	Saline	121.2 (±36.1)
	Exenatide (10 *μ*g/kg)	111.3 (±14.7)

Wfs1-deficient	Saline	110.4 (±12.7)
	Exenatide (10 *μ*g/kg)	103.9 (±17.7)

Glucagon was measured sixty minutes after the treatment. Two-way ANOVA was used to test for statistical differences. Data are mean ± SEM. *n* = 8 in all groups.
